# Hand grip strength variability during serial testing as an entropic biomarker of aging: a Poincaré plot analysis

**DOI:** 10.1186/s12877-020-1419-1

**Published:** 2020-01-13

**Authors:** Elena Ioana Iconaru, Constantin Ciucurel

**Affiliations:** Department of Medical Assistance and Physical Therapy, University of Pitesti, Pitesti, Romania

**Keywords:** Hand grip strength, Nonlinear dynamics, Time series, Poincaré plot, Entropy, Aging

## Abstract

**Background:**

The Poincaré plot method can be used for both qualitative and quantitative assessment of self-similarity in usually periodic functions, hence the idea of applying it to the study of homeostasis of living organisms. From the analysis of numerous scientific data, it can be concluded that hand functionality can be correlated with the state of the human body as a biological system exposed to various forms of ontogenetic stress.

**Methods:**

We used the Poincaré plot method to analyze the variability of hand grip strength (HGS), as an entropic biomarker of aging, during 60 repetitive tests of the dominant and nondominant hand, in young and older healthy subjects. An observational cross-sectional study was performed on 80 young adults (18–22 years old, mean age 20.01 years) and 80 older people (65–69 years old, mean age 67.13 years), with a sex ratio of 1:1 for both groups. For statistical analysis, we applied univariate descriptive statistics and inferential statistics (Shapiro–Wilk test, Mann–Whitney U-test for independent large samples, with the determination of the effect size coefficient r, and simple linear regression. We calculated the effect of fatigue and the Poincaré indices SD_1_, SD_2_, SD_1_/SD_2_ and the area of the fitting ellipse (AFE) for the test values of each subject.

**Results:**

The analysis of the differences between groups revealed statistically significant results for most HGS-derived indices (*p* ≤ 0.05), and the magnitude of the differences indicated, in most situations, a large effect size (*r* > 0.5). Our results demonstrate that the proposed repetitive HGS testing indicates relevant differences between young and older healthy subjects. Through the mathematical modeling of data and the application of the concept of entropy, we provide arguments supporting this new design of HGS testing.

**Conclusions:**

Our results indicate that the variability of HGS during serial testing, which reflects complex repetitive biomechanical functions, represents an efficient indicator for differentiation between young and older hand function patterns from an entropic perspective. In practical terms, the variability of HGS, evaluated by the new serial testing design, can be considered an attractive and relatively simple biomarker to use for gerontological studies.

## Background

### The Poincaré plot as a tool for mathematical modeling of data

The Poincaré plot defines a computational graphic tool (scattergram-type plot) for Cartesian representation of a series of data with dynamic nonlinear evolution. The application of the method refers, in particular, to the study of the time evolution of nonlinear phenomena that characterize open physical systems.

The Poincaré plot is based on the concept of constructing a return map (recurrence plot) of a time series of data and quantifies the recurrence, self-similarity, or periodicity of state variables of systems [1]. Practically, such analyses of the behavior of physical systems under different circumstances appeal to the concept of entropy. Entropy refers to the degree of disorder that characterizes a physical system, which can be appreciated in terms of the randomness and predictability of its evolution over time. Thus, greater entropy implies an increased randomness and a lower system order [2].

By analysing the geometric characteristics of the graphs plotted by the Poincaré method, it is possible to extract quantitative variables that help to characterize the dynamics of the systems taken into account [3]. Additionally, through complex statistical calculations, correlations across multiple spatiotemporal scales can be determined, which help to identify fractal dynamics [4]. Overall, using the proposed method, it is possible to achieve an entropy-based analysis of complex time series of data.

In mathematics, the Poincaré plot consists of a geometrical representation in the Cartesian plane of a time data string of the type x_1_, x_2_, x_3_, …, x_n_. Each value x_i_, where i = 1 … .n, refers to a time-based state of nonlinear evolution of a defined characteristic of a system. The return map of the data includes a plot for the following pairs of coordinates: (x_1_, x_2_), (x_2_, x_3_), … (x_i_, x_i + 1_)..., (x_n-1_, x_n_). Usually, the resulting plot is an ellipse, which can provide two important descriptors for a quantitative analysis of the dynamics of the time series data: SD_1_ and SD_2_, which in fact represent the minor and the major axes of the ellipse, respectively [5].

SD_1_ and SD_2_ are calculated through formulas that incorporate the standard deviation of the time series. Practically, SD_1_ represents the standard deviation (dispersion) of the distances of points from the major axis of the ellipse and indicates the short-term variability of the time series of data. SD_2_ represents the standard deviation (dispersion) from the minor axis of the ellipse and indicates the long-term variability of the time series of data [6].

The formulas for the two parameters are the following [5–7]:

$$ \mathrm{SD}1=\frac{\sqrt{2}}{2}\ast SD\left({x}_n-{x}_{n+1}\right) $$

$$ \mathrm{SD}2=\sqrt{2 SD{\left({x}_n\right)}^2-\frac{1}{2} SD{\left({x}_n-{x}_{n+1}\right)}^2} $$

where SD (x_n_ – x_n + 1_) represents the standard deviation of the time series x_n_ – x_n + 1_ and SD (x_n_) the standard deviation of the time series x_n_.

Another statistical indicator for the analysis of dynamic data evolution is the ratio SD_1_/SD_2_, which measures the relative balance between short- and long-term variabilities of the time series of data [8]. For this reason, SD_1_/SD_2_ reflects the clarity and linearity of the scatter pattern [9]. For statistical analysis, the area of the fitting ellipse (AFE) has been proposed, which is calculated by the formula AFE = *π* ∗ *SD*_1_ ∗ *SD*_2_ [7].

The utility of the Poincaré plot is justified by the fact that the initial qualitative analysis of a nonlinear distribution of data, by tracing the ellipse, can be transformed into a quantitative analysis in terms of linear statistics by calculating SD_1_ and SD_2_.

### Applications of Poincaré plot in the analysis of the biological systems dynamics

Because living organisms can be assimilated to open biological systems, various forms of their entropic behaviors have been proposed from the perspective of particular dynamic physiological parameters [10].

In this regard, the Poincaré plot has proven to be widely applicable in the field of human biology, for scale entropy analysis of complex physiologic time series [11] and for control systems involved in their complex regulation [12].

Traditionally, Poincaré plots have been used to represent biomedical signals such as heart rate and respiratory pattern variabilities [1]. Most studies of this kind are aimed at studying beat-to-beat heart rate variability in clinical circumstances. In this way, representations of R-R intervals can be extracted from serial electrocardiographic records.

The bivariate segmented Poincaré plot analysis, which is a variant derived from the classic one, is used to study the interactions between beat-to-beat RR intervals and its temporal enclosed systolic blood pressure time series [13].

At the respiratory level, the utility of the Poincaré plot analysis has been demonstrated for the exploration of the breathing pattern variability in terms of total breath duration, tidal volume, inspiratory time, expiratory time, and peak inspiratory flow [14, 15].

The Poincaré plot method was also proposed for the study of blood pressure variability [16], glucose variability from continuous glucose monitoring systems [7, 17], and the variability of some electroencephalographic paths [18, 19], polysomnography paths [9], plethysmography data [20], electromyography or electrohysterography data [6].

A new perspective for developing the Poincaré plot method has been projected for the purpose of comparing dynamical systems concepts and techniques for biomechanical analysis [4]. Thus, it is known that human movement implies entropy changes because body systems are exposed to various forms of stress constraints during locomotion.

In this way, the Poincaré plot analysis was proposed to characterize the mechanical time series fluctuations of gait. More explicitly, it quantified the foot clearance variability of patients in different clinical contexts (older people, neurodegenerative diseases, stroke etc.) [6, 21, 22] or other gait parameters (swing time, step length, and step width) [23]. For gait analysis through the Poincaré plot method, selected gait data signals (stride intervals) have been proposed for recording by some authors using force-sensitive resistors. These authors demonstrated that in healthy subjects, the parameters SD_1_ and SD_2_ are lower than in subjects with Parkinson’s disease and Huntington’s disease [6]. Another biomechanical study regarding the minimum foot clearance variability in the elderly during walking indicated increased irregularities and randomness in the gait patterns for individuals with balance problems and a history of falls, as an indication of loss of gait control mechanism [21]. Additionally, the Poincare indices SD_1_ (the short-term variability) and SD_2_ (the long-term variability) of the gait rhythm and the timing of the gait cycle can be readily employed to discern subjects with normal motor control from subjects with neurodegenerative disorder [22].

Another application of the Poincaré plot method is to analyze the center of pressure patterns during various sports-specific movements [24].

In conclusion, the Poincaré plot is used in clinical practice for the analysis of physiological time series, which are usually expressed as lengths, volumes, time intervals, pressures or concentrations of substances [7, 14, 16, 17, 23]. These parameters can be recorded during rest but also during exercise or other stress exposures [25, 26]. Although exercise induces fatigue, the parameters remain useful for the evaluation of human adaptation to physical effort.

### The perspective of applying the Poincaré plot for studying hand grip strength variability as an entropic biomarker of aging

From a thermodynamic point of view, the aging of an organism, as an open biological system, can be compared to a slow process with nonlinear dynamics of accumulation of molecular clutches and loss of complexity of repetitive structures (fractals), namely, the growth of whole-body entropy content [27]. Any dysfunction of the body or disease state also leads to increased entropy [28] and possibly to the intensification of the aging rate.

The Poincaré plot method can be used for both qualitative and quantitative assessment of self-similarity in usually periodic functions, hence the idea of the application to the study of homeostasis of living organisms. Therefore, taking into account the entropic nature of the homeostatic equilibrium [29], we consider innovative the proposal to use biomarkers of aging that reflect complex repetitive biomechanical functions that can be quantified by mathematical modeling.

In practice, hand grip strength (HGS) has wide usefulness for the standardized determination of the individual’s health and level of functionality, the impact of morbidity/disability, or the appreciation of the dynamics of the aging process [30]. In a recent study, we also demonstrated the applicability of HGS as a parameter for mathematical modeling for the study of the process of aging [31].

Classically, HGS is determined by limited procedures in terms of the number of repetitions (ordinarily, 1–3 repetitions), the highest recorded value of which is taken. Another proposed variant is the determination of the fatigue of the hand during isometric efforts of clamping of the dynamometer in various anatomical positions [32].

Very few studies have focused on repetitive tests of HGS, for example, for the analysis of endurance, muscle fatigue and hand weakness [33, 34].

## Methods

### Premises and aim of the study

From the analysis of numerous scientific data, it can be concluded that hand functionality can be correlated with the state of the human body as a biological system exposed to various forms of ontogenetic stress. Therefore, we considered using the Poincaré plot method to analyze the variability of HGS during repetitive tests for healthy subjects belonging to two age groups (young and old). By comparing the data series, we can draw conclusions on the dynamics of the normal aging process, the new test design having possible worth as an entropic biomarker of the aging process.

### Participants and type of study

An observational cross-sectional study was performed on two groups of healthy subjects. The first group consisted of 80 young adults aged 18–22 years (mean age 20.01 years), and the second group consisted of 80 older people aged 65–69 years (mean age 67.13 years). The sex ratio was 1:1 for both groups of subjects.

Young adults were selected from among the students of the University of Pitesti and older people from among community-dwelling people. All participants, at the time of testing, had a good health status, were completely autonomous in activities of daily living (ADL) and did not have a significant pathological history (chronic or acute pathology of the upper limbs, recent injuries, arthritis, neuromuscular disorders or other medical conditions that could interfere with HGS). Subjects were untrained individuals with previous experience with light or moderate leisure physical activity. For each individual, we recorded the informed consent to participate in the study in accordance with the ethics of research on human subjects. Ethics approval was obtained from the Ethics Committee of the Research Center for Promoting Excellence in Professional Training, University of Pitesti (reference number 836/20.04.2018).

### Data acquisition

The study was performed over 9 months in 2018. Through anamnesis, we collected personal information from each participant regarding the health status and previous medical history. Next, for each of them, we assessed the HGS alternatively for both hands using a hydraulic hand dynamometer (Saehan model, MSD Europe bvba, Belgium). The results were recorded in kilogram-force (kgf). This type of device has good references for clinical use, in terms of validity and reliability, similar to the hydraulic Jamar dynamometer [35].

According to classical recommendations for obtaining maximal HGS results, participants sat on a chair, with feet flat on the floor, with the elbow flexed at 90°, the forearm in a neutral position and the wrist between 0° and 30° of extension [36]. Basically, the goal was to choose a position as comfortable as possible for each subject within the mentioned interval. This fact is in accordance with the recommendations for optimal dynamometer handle position to assess maximal isometric HGS in epidemiological studies and with the recommendations of the American Society of Hand Therapist [36].

As a standard procedure, the dynamometer’s base must be applied on the first metacarpal bone (inside the palm), with the handle in contact with half of the last four fingers. To fit each individual’s palm size, the dynamometer’s handle was adjusted for each subject (the handle allows fine adjustment, from 3.8 cm to 8.9 cm, in 5 steps).

For each hand, 60 successive tests were performed over an 8-min interval in the form of repeated maximal isometric efforts, with a duration of 3 s each, followed by a relaxation phase of 5 s (when the result was read). No other body movement was allowed during testing. The subjects were encouraged to reach the maximal results, this condition requiring a high concentration on the task. Our protocol was designed by starting from the classic recommendations for clinical determination of HGS regarding the duration of maximal isometric effort [37]. For the determination of hand grip endurance with repetitive maximal isometric contractions, some authors have used 2.5 s for contractions and 1 s for rest time [38]. In our case, a higher value of the pause (5 s) was proposed to reduce muscle fatigue. For this reason, the selected number of 60 successive tests is relatively easy for healthy subjects to do and does not cause excessive fatigue.

To ensure a uniform test pace and to assist the subjects, we used a sensory multimedia support with visual and acoustic feedback. Hence, on a digital display (smartphone or tablet), we ran a .gif file to synchronize the testing phases with the rest in the form of a countdown timer. Initially, the dominant hand, considered to be the one preferred for ADL (writing), was tested. Next, the procedure was repeated for the nondominant hand after a 5-min break. Classically, a pause of at least 1 min is recommended between the two tests [37]. In our case, a 5-min extended pause between the measurements with the dominant and nondominant hands was selected to ensure that the fatigue from the measurement of the one hand did not influence the other hand.

Each subject performed a short familiarization trial (4–5 HGS contractions) 5 min before the main testing to familiarize themselves with the procedure. The testing started from the resting state, without a warm-up session. This was motivated by the fact that the increase in HGS due to warm-up could be clinically significant [39].

Common anthropometric measurements (body height, H; body mass, M) were also performed according to standard procedures. All assessments were completed in the morning, between 8 and 11 a.m.

### Outcomes and statistical analysis

Data series of HGS were grouped by sex, age (young, older people), and laterality (dominance of the hand). For statistical analysis, we applied univariate descriptive statistics and inferential statistics. Therefore, we initially calculated the mean HGS and the Poincaré indices SD_1_, SD_2_, SD_1_/SD_2_ and AFE for each subject and for each hand. Starting from the H and W values, we also calculated the body mass index (BMI) for each participant. We defined another parameter, the effect of fatigue, as an average decrease in HGS, as a percentage change between the first and the last evaluation in the set of 60 tests.

Next, we determined the mean and standard deviation (SD) for the young and older groups (men, women, men and women together). As examples, we plotted the Poincaré graphs for one subject of each group.

The data series was checked for normality by means of the Shapiro–Wilk test, and since all of the data presented a nonnormal distribution, we applied the Mann–Whitney U-test for independent large samples (*N* > 20) to check the statistical significance of the differences between groups (calculation of the U and z values).

To evaluate the magnitude of the difference between groups, we determined the effect size coefficient r with the following formula: r $=\frac{\mathrm{z}}{\surd \mathrm{n}}$, where n is the number of subjects [40]. The interpretation of the r coefficient is as follows: values greater than 0.5 denote a large effect size, values in the range of 0.3–0.5 indicate a medium effect size, and values smaller than 0.1 indicate a small effect size [41]. Finally, we run a simple linear regression model for the analyzed variables.

## Results

The results are centralized as descriptive statistical indicators in Tables 1 and 2. It is noteworthy that the statistical indicators for HGS, in all situations, could be included in normative data according to sex and laterality for the concerned age groups [42, 43]. Interpretations should be made by noting that the mean values of HGS for the study groups refer to the series of values obtained by 60 successive measurements for each participant’s hand.

**Table 1:** Statistic indicators for investigated parameters in the young groups

Variable	Age years	M kg	H cm	BMI kg/m^2^	HGS kgf		Fatigue %		SD_1_ kgf		SD_2_ kgf		SD_1_/SD_2_		AFE kgf^2^	
Men (*n* = 40)																
					D	ND	D	ND	D	ND	D	ND	D	ND	D	ND
Mean	20.08	66.70	175.34	21.65	46.85	43.39	15.89	16.87	1.18	1.13	3.36	3.51	0.36	0.36	13.60	13.31
SD	1.12	7.41	6.71	1.50	6.09	5.63	7.68	8.54	0.42	0.36	1.32	1.52	0.10	0.14	12.08	9.95
Women (*n* = 40)																
					D	ND	D	ND	D	ND	D	ND	D	ND	D	ND
Mean	19.95	55.11	164.55	20.33	26.07	23.38	24.53	26.70	1.19	1.14	3.44	3.25	0.36	0.37	13.07	11.73
SD	1.22	6.30	6.13	1.73	5.00	4.50	8.52	10.43	0.37	0.30	0.79	0.71	0.14	0.13	5.62	4.28
Men and women (*n* = 80)																
					D	ND	D	ND	D	ND	D	ND	D	ND	D	ND
Mean	20.01	60.90	169.94	20.99	36.46	33.38	20.21	21.78	1.19	1.13	3.40	3.38	0.36	0.36	13.34	12.52
SD	1.16	8.98	8.38	1.74	11.83	11.27	9.15	10.68	0.39	0.33	1.08	1.18	0.12	0.13	9.36	7.65

*M* body mass, *H* body height, *BMI* body mass index, *HGS* hand grip strength, *D* dominant, *ND* nondominant, *SD* standard deviation, *AFE* area of the fitting ellipse, *n* number of subjects

**Table 2:** Statistic indicators for investigated parameters in the older groups

Variable	Age years	M kg	H cm	BMI kg/m^2^	HGS kgf		Fatigue %		SD_1_ kgf		SD_2_ kgf		SD_1_/SD_2_		AFE kgf^2^	
Men (*n* = 40)																
					D	ND	D	ND	D	ND	D	ND	D	ND	D	ND
Mean	67.30	79.20	173.03	26.50	31.44	27.68	34.74	38.61	1.56	1.50	6.13	6.03	0.29	0.28	29.65	28.42
SD	1.26	9.18	7.72	3.02	7.35	6.80	15.86	12.45	0.26	0.27	1.82	1.91	0.17	0.13	9.01	9.96
Women (*n* = 40)																
					D	ND	D	ND	D	ND	D	ND	D	ND	D	ND
Mean	66.95	67.11	159.56	26.39	19.64	17.10	48.09	53.84	1.63	1.58	5.38	5.05	0.35	0.34	27.41	25.17
SD	1.34	8.02	6.75	3.02	3.52	3.17	12.72	7.82	0.26	0.21	1.52	1.25	0.17	0.11	8.26	7.42
Men and women (*n* = 80)																
					D	ND	D	ND	D	ND	D	ND	D	ND	D	ND
Mean	67.13	73.15	166.29	26.45	25.54	22.39	41.42	46.23	1.60	1.54	5.76	5.54	0.32	0.31	28.53	26.80
SD	1.31	10.50	9.89	3.00	8.25	7.49	15.78	12.86	0.26	0.25	1.71	1.68	0.17	0.12	8.66	8.87

*M* body mass, *H* body height, *BMI* body mass index, *HGS* hand grip strength, *D* dominant, *ND* nondominant, *SD* standard deviation, *AFE* area of the fitting ellipse, *n* number of subjects

Clearly, during HGS serial testing, the HGS values tended to decrease gradually due to the phenomenon of muscle fatigue. Therefore, the maximum values during these successive measurements can be considered representative for each subject as the reference level of HGS. At the same time, the mean values of HGS indicate the fatigue over time and the adaptability to serial testing for the investigated subjects.

For the inferential statistical analysis, a nonparametric test (the Mann-Whitney U-test) was applied to compare the differences between data series with a nonnormal distribution. In fact, the existence of this type of distribution was expected, considering the temporal nonlinearity of the evolution of the parameters derived from the HGS values (Tables 3, 4 and 5).

**Table 3:** Comparison of recorded parameters between young and older men groups

Parameters	Young men (*n* = 40), mean ± SD	Older men (*n* = 40), mean ± SD	U	z	p	r
HGS, D (kgf)	46.85 ± 6.09	31.44 ± 7.35	100	6.73	≤ 0.05	1.06
Fatigue, D (%)	15.89 ± 7.68	34.74 ± 15.86	203	−5.74	≤ 0.05	− 0.91
SD_1_, D (kgf)	1.18 ± 0.42	1.56 ± 0.26	291.5	−4.89	≤ 0.05	−0.77
SD_2_, D (kgf)	3.36 ± 1.32	6.13 ± 1.82	191	−5.86	≤ 0.05	−0.93
SD_1_/SD_2_, D	0.36 ± 0.10	0.29 ± 0.17	375	4.08	≤ 0.05	0.65
AFE, D (kgf^2^)	13.60 ± 12.08	29.65 ± 9.01	145	−6.30	≤ 0.05	−1
HGS, ND (kgf)	43.39 ± 5.63	27.68 ± 6.80	86.5	6.86	≤ 0.05	1.08
Fatigue, ND (%)	16.87 ± 8.54	38.61 ± 12.45	162.5	− 6.13	≤ 0.05	− 0.97
SD_1_, ND (kgf)	1.13 ± 0.36	1.50 ± 0.27	270	−5.10	≤ 0.05	− 0.81
SD_2_, ND (kgf)	3.51 ± 1.52	6.03 ± 1.91	240.5	−5.38	≤ 0.05	−0.85
SD_1_/SD_2_, ND	0.36 ± 0.14	0.28 ± 0.13	491.5	2.96	≤ 0.05	0.47
AFE, ND (kgf^2^)	13.31 ± 9.95	28.42 ± 9.96	179.5	−5.97	≤ 0.05	−0.94

*HGS* hand grip strength, *D* dominant, *ND* nondominant, *SD* standard deviation, *AFE* area of the fitting ellipse, *n* number of subjects, *U* Mann–Whitney’s U-test values, *z* scores, *p* thresholds of statistical significance, *r* effect size coefficient

**Table 4:** Comparison of recorded parameters between young and older women groups

Parameters	Young women (*n* = 40), mean ± SD	Older women (*n* = 40), mean ± SD	U	z	p	r
HGS, D (kgf)	26.07 ± 5.00	19.64 ± 3.52	226.5	5.51	≤ 0.05	0.87
Fatigue, D (%)	24.53 ± 8.52	48.09 ± 12.72	146	−6.29	≤ 0.05	−0.99
SD_1_, D (kgf)	1.19 ± 0.37	1.63 ± 0.26	235	− 5.43	≤ 0.05	−0.86
SD_2_, D (kgf)	3.44 ± 0.79	5.38 ± 1.52	203	−5.74	≤ 0.05	−0.91
SD_1_/SD_2_, D	0.36 ± 0.14	0.35 ± 0.17	657	1.37	–	–
AFE, D (kgf^2^)	13.07 ± 5.62	27.41 ± 8.26	122.5	−6.51	≤ 0.05	−1.03
HGS, ND (kgf)	23.38 ± 4.50	17.10 ± 3.17	182	5.94	≤ 0.05	0.94
Fatigue, ND (%)	26.70 ± 10.43	53.84 ± 7.82	27	−7.43	≤ 0.05	−1.17
SD_1_, ND (kgf)	1.14 ± 0.30	1.58 ± 0.21	206.5	−5.71	≤ 0.05	−0.90
SD_2_, ND (kgf)	3.25 ± 0.71	5.05 ± 1.25	187	−5.89	≤ 0.05	−0.93
SD_1_/SD_2_, ND	0.37 ± 0.13	0.34 ± 0.11	677	1.18	–	–
AFE, ND (kgf^2^)	11.73 ± 4.28	25.17 ± 7.42	72	−7.00	≤ 0.05	−1.11

*HGS* hand grip strength, *D* dominant, *ND* nondominant, *SD* standard deviation, *AFE* area of the fitting ellipse, *n* number of subjects, *U* Mann–Whitney’s U-test values, *z* scores, *p* thresholds of statistical significance, *r* effect size coefficient

**Table 5:** Comparison of recorded parameters between young and older groups

Parameters	Young group (*n* = 80), mean ± SD	Older group (*n* = 80), mean ± SD	U	z	p	r
HGS, D (kgf)	36.46 ± 11.83	25.54 ± 8.25	1472	5.90	≤ 0.05	0.66
Fatigue, D (%)	20.21 ± 9.15	41.42 ± 15.78	806.5	−8,17	≤ 0.05	−0.91
SD_1_, D (kgf)	1.19 ± 0.39	1.60 ± 0.26	1090	−7.20	≤ 0.05	−0.80
SD_2_, D (kgf)	3.40 ± 1.08	5.76 ± 1.71	816	−8.13	≤ 0.05	−0.91
SD_1_/SD_2_, D	0.36 ± 0.12	0.32 ± 0.17	2015	4.04	≤ 0.05	0.45
AFE, D (kgf^2^)	13.34 ± 9.36	28.53 ± 8.66	551.5	−9.04	≤ 0.05	−1.01
HGS, ND (kgf)	33.38 ± 11.27	22.39 ± 7.49	1379.5	6.21	≤ 0.05	0.69
Fatigue, ND (%)	21.78 ± 10.68	46.23 ± 12.86	520	−9.14	≤ 0.05	−1.02
SD_1_, ND (kgf)	1.13 ± 0.33	1.54 ± 0.25	989	−7.54	≤ 0.05	−0.84
SD_2_, ND (kgf)	3.38 ± 1.18	5.54 ± 1.68	889.5	−7.88	≤ 0.05	−0.88
SD_1_/SD_2_, ND	0.36 ± 0.13	0.31 ± 0.12	2361.5	2.86	≤ 0.05	0.32
AFE, ND (kgf^2^)	12.52 ± 7.65	26.80 ± 8.87	533.5	−9.10	≤ 0.05	−1.02

*HGS* hand grip strength, *D* dominant, *ND* nondominant, *SD* standard deviation, *AFE* area of the fitting ellipse, *n* number of subjects, *U* Mann–Whitney’s U-test values, *z* scores, *p* thresholds of statistical significance, *r* effect size coefficient

The analysis of the differences between groups revealed statistically significant results for most HGS-derived indices (*p* ≤ 0.05), with the exception of the ratio SD_1_/SD_2_ (for the dominant and nondominant hands), when comparing the groups of young women and older women.

In the case of statistically significant results, the magnitude of the differences between the data series indicated, in most situations, a large effect size (*r* > 0.5). There were also three distinct situations in which the *r* values indicated a medium effect size: for the SD_1_/SD_2_ ratio of the nondominant hand when comparing the groups of young men and older men and for the SD_1_/SD_2_ ratio of the dominant and nondominant hands when comparing the groups of young and older subjects.

Next, we applied a standard linear analysis of the fatigue and the Poincaré parameters. The fact that the variables do not have a normal distribution is not an impediment, and linear regression remains a statistically sound technique in studies of large sample sizes [44]. For this reason, we applied the simple linear regression at the level of young and older groups (*n* = 80). The results of simple linear regression analysis which was conducted to determine the effect of fatigue (the predictor variable) on the parameters measured (the outcome variables) are presented in Table 6.

**Table 6:** Results of the simple linear regression analysis for the effect of fatigue on recorded parameters in young and older groups

Variable	*R*	R square	SE	*F*	*p*	Regression equation
Young group (*n* = 80)						
HGS, D	0.62	0.38	9.36	48.24	0.001	y = −0.80 *x + 52.61
SD_1_, D	0.22	0.05	0.39	4.04	0.048	y = −0.01 *x + 0.99
SD_2_, D	0.64	0.41	0.83	54.52	0.001	y = 0.08 *x + 1.87
SD_1_/SD_2_, D	0.37	0.13	0.11	12.16	0.001	y = −0.01 *x + 0.46
AFE, D	0.50	0.25	8.14	26.58	0.001	y = 0.52 *x + 2.92
HGS, ND	0.53	0.28	9.60	30.77	0.001	y = −0.56 *x + 45.61
SD_1_, ND	0.02	0.001	0.33	0.05	–	y = 0.001 *x + 1.12
SD_2_, ND	0.51	0.26	1.03	26.88	0.001	y = 0.06 *x + 2.16
SD_1_/SD_2_, ND	0.47	0.22	0.12	21.55	0.001	y = −0.01 *x + 0.49
AFE, ND	0.37	0.14	7.16	12.24	0.001	y = 0.26 *x + 6.77
Older group (*n* = 80)						
HGS, D	0.71	0.51	5.81	81.21	0.001	y = −0.37 *x + 41
SD_1_, D	0.30	0.09	0.25	7.99	0.006	y = −0.01 *x + 1.80
SD_2_, D	0.53	0.28	1.46	30.69	0.001	y = 0.06 *x + 3.37
SD_1_/SD_2_, D	0.68	0.46	0.13	67.68	0.001	y = −0.01 *x + 0.63
AFE, D	0.40	0.16	7.97	15.26	0.001	y = 0.22 *x + 19.34
HGS, ND	0.81	0.65	4.43	147.52	0.001	y = −0.47 *x + 44.15
SD_1_, ND	0.12	0.02	0.24	1.24	–	y = −0.002 *x + 1.65
SD_2_, ND	0.26	0.07	1.63	5.80	0.018	y = 0.03 *x + 3.96
SD_1_/SD_2_, ND	0.39	0.15	0.11	13.97	0.001	y = −0.004 *x + 0.47
AFE, ND	0.16	0.03	8.81	2.07	–	y = 0.11 *x + 21.66

*HGS* hand grip strength, *D* dominant, *ND* nondominant, *SD* standard deviation, *AFE* area of the fitting ellipse, *n* number of subjects, *R* coefficient of correlation, *R square* coefficient of determination, *SE* standard error, *F* value of F-test for overall significance, *p* thresholds of statistical significance, *x* the predictor variable (fatigue), *y* the outcome variable

We found that our regression model is statistically significant in most cases. Therefore, a certain part of the variability of the analyzed parameters is explained by the appearance of the fatigue effect.

## Discussion

At present, biological aging can best be quantified through compound aging scores, and HGS is used as a singular indicator in a classic manner as an approximate predictor of longevity, disability, frailty and/or morbidity [45, 46].

To our knowledge, the present study is the first to consider the biomechanical analysis of repetitive HGS testing through the Poincaré plot method to investigate the aging process in terms of entropy. Although there are a few studies that analyze the HGS variation during serial tests, these included relatively small series of tests, on the order of 10–15 repetitions. In most cited cases, such testing protocols have proven to be reliable [34, 47, 48]. The innovation of our study lies in designing an investigation of the HGS variability during repetitive tests for young and older subjects by using mathematical modeling of the recorded data. Thus, for each participant, a significant number of repetitions of the test was performed (60 successive tests), feasible for data analysis through the Poincaré plot method [49].

Following the dynamics of the HGS values during the serial testing of the subjects, a series of relevant observations can be made. Interestingly, in several cases, the maximum values recorded for HGS were not reached during the first 5 tests in the 60-test series. Hence, the classic recommendation to perform 3 HGS determinations, of which the highest value is selected [50, 51], is not always fully relevant to this biomechanical parameter.

Additionally, for the proposed protocol, muscle fatigue occurred in an atypical way. Thus, a nonlinear descendent trend in HGS values was observed over time, and the HGS minimum values were sometimes recorded prior to the last 5 tests. In addition, there were subjects with atypical individual evolution during the serial testing. In other words, such situations cannot be excluded, a fact already confirmed by other authors [52].

For our testing protocol, we considered the occurrence of muscle fatigue as an inherent phenomenon that decreases muscle strength. The nonlinear character of the HGS during serial testing, however, indicates the variability of the efficiency of the regulation system of the imposed motor task. Essentially, muscle fatigue, as a physiological process, involves complex systems of neuromotor and metabolic regulation and might be measured as the entropy level [53]. Additionally, fatigue is influenced by the aging process, and older adults seem to be more susceptible to fatigue during high-speed dynamic contractions [54].

Overall, in the young men group, we found a fatigue effect of 15.89% for the dominant hand and 16.87% for the nondominant hand. For the young women group, the mean values were 24.53 and 26.7%, respectively. In the older men group, the recorded mean values were 34.74 and 38.61%, and in the older women group, they were 48.09 and 53.84%, respectively. Clearly, the muscular fatigue of the hand during testing was of a general nature among the subjects, being more noticeable in women, regardless of age. Moreover, the described phenomenon was much broader in older people than young subjects, which could be explained by the decline in neuromotor functions associated with the aging process.

Analyzing the values for R squared from Table 6, we observe, as expected, that the fatigue influences more strongly the mean HGS in all situations. In the case of the parameters derived from the Poincaré plot method, there are similarities, but also differences in the relations with the fatigue, as the predictor variable. Overall, the effect of fatigue is more evident at the level of SD_2_, SD_1_/SD_2_, and AFE and lower at the level of SD_1_, for both hands and age groups. Things are plausible because SD_2_ reflects the long-term variability and SD_1_ the short-term variability. The fatigue effect mainly induces the change in the long-term variability of HGS. Also, the maximum effect of fatigue was identified in the case of SD_1_/SD_2_ for the dominant hand of older people, when 46% of the variation of the parameter is explained by the regressor fatigue.

The serial testing of the HGS determined the appearance of nonlinear oscillations of the motor performance of the subjects’ hands, with a decreasing tendency. This phenomenon can be explained by the gradual appearance of muscle fatigue and by the variability of the efficiency of the neuromotor and metabolic control mechanisms. Moreover, muscle fatigue itself reflects a failure of neuromuscular regulation and muscle metabolic adaptation in response to exercise [55]. In other words, a complex motor task (such as HGS) can be performed successively over time with a certain constant rhythm as long as the processes described above involve adaptive capabilities of the movement system. During aging, the human body, as an open biological system, manifests limitations of metabolic and functional adaptation to stress factors, which is also reflected in the decline in skeletal muscle performance and physical performance [56].

The decrement in HGS performance has been reported in other studies that have focused on identifying HGS endurance during dynamic evaluations [34, 48, 57]. Other authors consider that, at least for repeated series of 10 consecutive HGS tests, the performance in the test is quite stable over trials, although there is the expected fatigue effect, especially for men [47]. However, the literature on this topic is inconsistent when it comes to small series of repeat tests [50]. Obviously, the gradual decrease in HGS by successive testing is generally conditioned by the number of repetitions and by the intervals between them [58].

A dynamic protocol, for example, of 12 repeated maximal repetitions, is recommended for the evaluation of dynamic HGS endurance, using as reliable indicators the maximal HGS and the percentage change [59]. In our study, the objective of the data series analysis was to collaterally target the evaluation of HGS endurance, which was anticipated to occur in the proposed protocol. Instead, from an entropic perspective, we found that the mechanical time-series fluctuations of the HGS differed between the young group and the older group, regardless of sex. The obtained indices of variability, highlighted by the Poincaré plot method, were significantly higher for the older group than for the young group overall, and this difference held in the subgroups of men and women (*p* ≤ 0.05).

**Fig. 1:**
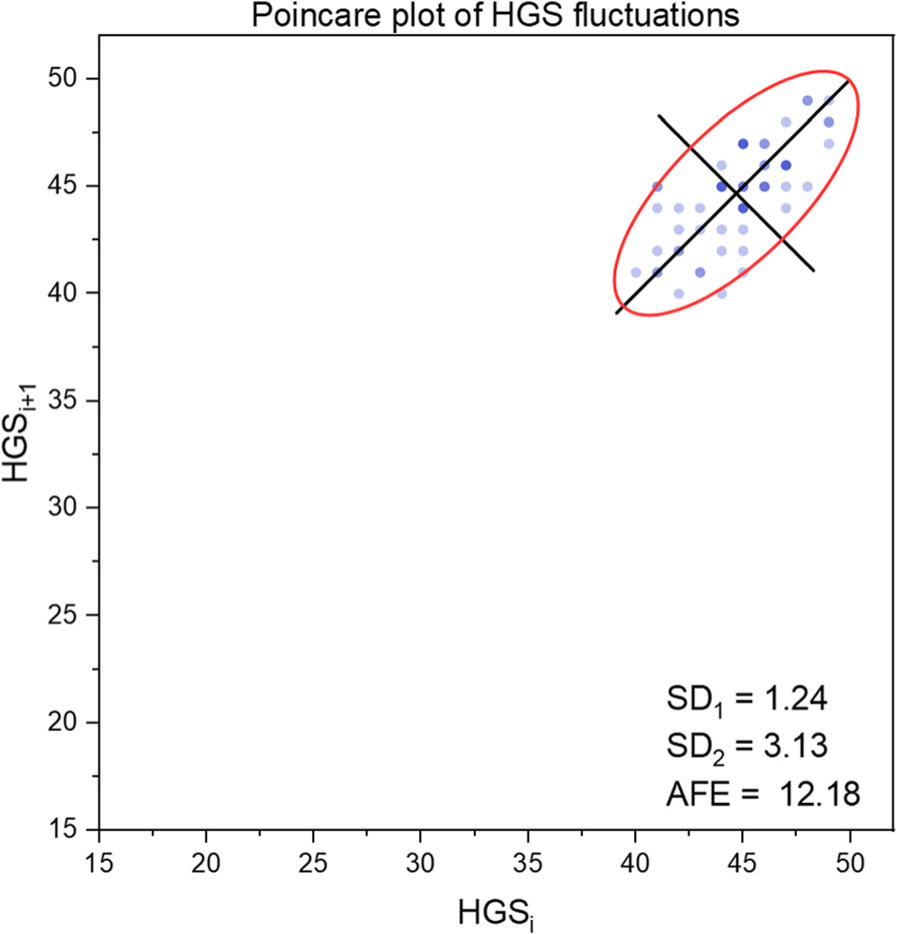
Typical sample of Poincaré plot of HGS fluctuations (dominant hand), for a young subject (man). HGS: hand grip strength; AFE = area of the fitting ellipse

**Fig. 2:**
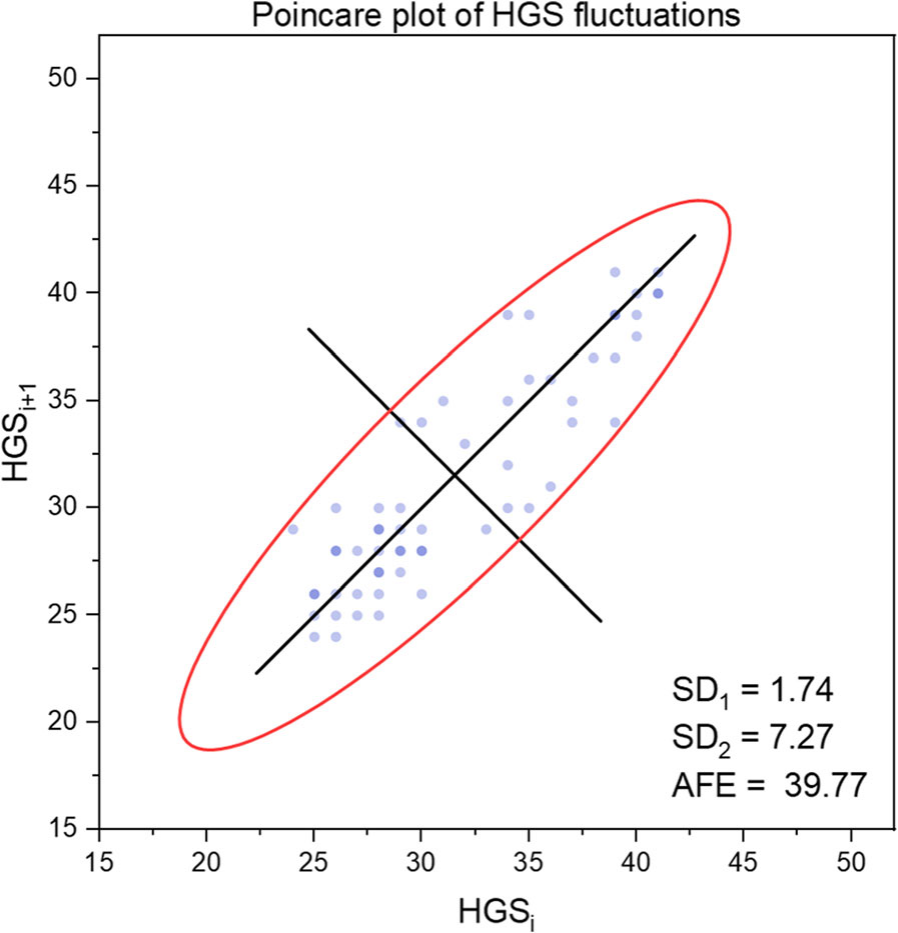
Typical sample of Poincaré plot of HGS fluctuations (dominant hand) for an older subject (man). HGS: hand grip strength; AFE = area of the fitting ellipse

The results can be interpreted in a more refined manner by taking into account one at a time the SD_1_, SD_2_, SD_1_/SD_2_ and AFE indices. As examples, the pattern of HGS fluctuations (dominant hand) of representative subjects from the groups of young and older subjects can be observed in Figs. 1 and 2. The subjects were chosen according to the mean values of the SD_1_ and SD_2_ indices of the groups.

The decrease in HGS force for the young participant was 18.37% and for the old participant was 41.46%. Clearly, muscular fatigue was present in both cases and affected the old subjects more but appeared to be a nonlinear phenomenon. From the entropic perspective, fatigue was associated with greater variability of HGS in older subjects. As a consequence, the values SD_1_, SD_2_, and AFE were significantly smaller for the younger subjects than for the old ones. Mainly, the mechanisms of neuromuscular and metabolic control involved in supporting the process of achieving the imposed complex motor task, which also implies the onset of muscle fatigue, are more deficient in older people.

At the group level, the analysis of SD_1_ and SD_2_ indices argues that performance in HGS is more stable in young subjects, despite the expected fact that muscle fatigue occurred for all participants. In fact, the more noticeable changes occurred in SD_2_ than in SD_1_, which reflects the long-term variability of the time series of data.

The differences between the means of the SD_1_/SD_2_ ratio were less extensive and more difficult to interpret, but they can be presented in terms of the relative uniformity of the balance between short- and long-term variabilities of the time series of data.

Finally, significant differences between groups of young and older people, regardless of sex, were found in the AFE values. Generally, an elliptic plot indicates an oscillating system, and as the ellipse area gets smaller (the data become more concentrated), the greater the stability of the system and the lower its entropy is [60]. Conversely, as the ellipse area becomes larger (the data are more scattered), the tendency to irregularity is more pronounced, and the system has more variable behavior and higher entropy.

We demonstrated that the AFE mean values of the older people were higher than those of young individuals, both for men and women, as well as for the combined groups of men and women. In other words, the behavior of the organism during HGS testing, as a biomechanical system, signifies a lower level of entropy in young subjects compared to the older subjects. This implies the existence of more marked features of repeatability, uniformity, predictability and homogeneity of the data series in young adults, which means the system is functioning in a regime of maximum efficiency and resistance. Instead, the manifestation of the aging process involves a reduction in system performance in terms of focus on the task, coordination of movements, steady rhythm of HGS development, and earlier occurrence of muscle fatigue. In brief, overall, the degree of disorder of these elements in the context of achieving the required task signifies higher entropy in the older organism.

From the entropic perspective, hand sensorimotor function has been studied by other authors, for example, in the case of diabetes mellitus. In these patients, there was a reduced force structural complexity of the hand (lower approximate entropy values) than in healthy subjects when producing grip force during submaximal force production tasks [61]. Additionally, in other pathologies, such as stroke, the increased force variability constitutes a hallmark of arm disabilities [62]. In multiple sclerosis, the fatigability of repeated hand grip strength is correlated with disease progression [57], and aging causes an increase in the motor output variability during rapid discrete isometric contractions [63].

Clearly, in terms of entropy, the pathological and aging systems are less complex than healthy systems, and this finding has been attributed to the degraded physiological control processes [64]. The interpretation of our results converges to the same idea, showing that the proposed repetitive HGS testing evidences statistically significant differences between young and older healthy people. Through the mathematical modeling of data and the application of the concept of entropy, we provide arguments supporting this new design of HGS testing.

### Limitations of the study

This study has some limitations. First, the proposed test is more complex than it seems, and the collected results can be considered significant only to the extent that the premises of rigorous testing are provided. Practically, interindividual differences can be reported regarding the participant’s involvement in meeting the requirements of the testing methodology. Second, the number of successive tests and proposed timeframes can be resized, depending on the objectives of the study and the resources of the subjects being tested. Last but not least, we did not consider variables such as the participants’ comorbidities or lifestyle that could interfere with HGS assessment. An extrapolation of the experimental design for other types of individuals, with functional limitations, should be reconsidered through a reduction of the number of serial tests and/or an increase in the pauses between contractions. In the future, by studying large samples of participants in several age groups and taking into account composite variables, the possible procedural errors can be mitigated.

## Conclusions

Our results indicate that the variability of HGS during serial testing represents an efficient indicator for differentiation between young and older hand function patterns from an entropic perspective. The fatigue effect is present at the level of the dynamics of the recorded entropic parameters and should be considered as an important factor for data interpretation. Obviously, aging is manifested in the level of hand functionality through the time performance fluctuations of the task of achieving repetitive HGS. This fact can be interpreted in the sense of the functioning of the senescent organism as a system characterized by irregularity and oscillation, and therefore with the tendency to increase entropy.

In practical terms, the variability of HGS, evaluated by the new serial testing design, can be considered an attractive and relatively simple biomarker to use for gerontological studies. The method of analysis of HGS time series according to the Poincaré plot technique has the potential for development and refining in the future for extensive clinical applications as well as for the delimitation of normal and pathological aging.

## Data Availability

The datasets used and/or analyzed during the current study are available from the corresponding author upon reasonable request.

## Abbreviations

AFE: Area of the fitting ellipse
BMI: Body mass index
D: Dominant
H: Body height
HGS: Hand grip strength
L: Left
M: Body mass
n: number of subjects
ND: Non-dominant
R: Right
SD: Standard deviation
